# An Electrochemical Biosensor for the Detection of Pulmonary Embolism and Myocardial Infarction

**DOI:** 10.3390/bios14080386

**Published:** 2024-08-09

**Authors:** Yaw-Jen Chang, Fu-Yuan Siao, En-Yu Lin

**Affiliations:** 1 Department of Mechanical Engineering, Chung Yuan Christian University, Chung Li District, Taoyuan City 320314, Taiwan; 2 Department of Emergency and Critical Care Medicine, Changhua Christian Hospital, Changhua 50006, Taiwan

**Keywords:** pulmonary embolism, myocardial infarction, factor VIII (FVIII), cardiac troponin I (cTnI), silk fibroin (SF)

## Abstract

Due to the clinical similarities between pulmonary embolism (PE) and myocardial infarction (MI), physicians often encounter challenges in promptly distinguishing between them, potentially missing the critical window for the correct emergency response. This paper presents a biosensor, termed the PEMI biosensor, which is designed for the identification and quantitative detection of pulmonary embolism or myocardial infarction. The surface of the working electrode of the PEMI biosensor was modified with graphene oxide and silk fibroin to immobilize the mixture of antibodies. Linear sweep voltammetry was employed to measure the current-to-potential mapping of analytes, with the calculated curvature serving as a judgment index. Experimental results showed that the curvature exhibited a linear correlation with the concentration of antigen FVIII, and a linear inverse correlation with the concentration of antigen cTnI. Given that FVIII and cTnI coexist in humans, the upper and lower limits were determined from the curvatures of a set of normal concentrations of FVIII and cTnI. An analyte with a curvature exceeding the upper limit can be identified as pulmonary embolism, while a curvature falling below the lower limit indicates myocardial infarction. Additionally, the further the curvature deviates from the upper or lower limits, the more severe the condition. The PEMI biosensor can serve as an effective detection platform for physicians.

## 1. Introduction

The symptoms of chest pain can vary from sharp stabbing sensations to dull aches [1,2]. Among the five common or dangerous causes of chest pain are gastroesophageal reflux, neuromuscular pain, pulmonary embolism (PE), aortic dissection, and acute myocardial infarction (MI) [3,4]. However, the most life-threatening origins typically involve the heart or lungs. PE presents as a severe condition characterized by sudden shortness of breath and chest pain [5,6,7,8]. It occurs when blood clots, most commonly originating from lower extremity venous thromboembolism, travel to the pulmonary artery through the bloodstream, obstructing blood vessels in the lungs. While PE is a serious yet treatable ailment, untreated cases can result in heart or lung damage, and even death. It has been noted that one-third of PE patients succumb to the condition before diagnosis and treatment. Conversely, MI arises from the occlusion of a coronary artery, typically due to atherosclerosis—a buildup of plaque consisting of deposits, cholesterol, and other substances [9,10,11]. When these arteries become obstructed, blood flow returning to the heart is impeded or greatly reduced. Consequently, cardiomyocytes supplied by these arteries are deprived of oxygen and nutrients, leading to permanent damage to the heart tissue. This irreversible damage initiates approximately 30 min after the onset of blockage and manifests as acute and potentially fatal heart disease. Prompt treatment of MI is imperative.

The most commonly employed clinical tests for detecting PE include computed tomography (CT) scan, pulmonary angiogram, magnetic resonance imaging (MRI) of the legs or lungs, etc. Additionally, D-dimer, a small protein fragment present in the blood following clot degradation, has been utilized to assess various conditions such as deep vein thrombosis (DVT) and/or PE through methods like enzyme-linked immunosorbent assay (ELISA) or other assays [12,13]. For MI diagnosis, blood tests utilizing cardiac biomarkers like troponins are commonly employed to ascertain myocardial damage. Furthermore, electrocardiogram (ECG), echocardiography, and myocardial perfusion imaging are frequently used clinical diagnostic tools. However, these tests for PE and MI rely on costly and sophisticated instrumentation.

Despite the distinct etiologies of PE and MI, both conditions present clinical symptoms including chest pain, dyspnea, night sweats, and fainting. Owing to these similarities, instantaneous diagnosis by medical professionals becomes challenging, potentially leading to missed opportunities for timely emergency care, thereby increasing the risk of patient mortality. Therefore, when patients in the emergency room present with chest pain, accurate diagnosis and timely treatment are key to improving patient survival rates. To address this challenge, this study developed a biosensor named the PEMI biosensor, designed for the identification and quantitative detection of PE and MI. By detecting the coagulation factor VIII (FVIII) and cardiac troponin I (cTnI), this PEMI biosensor can differentiate between PE and MI using minimal sample volumes.

The elevation of FVIII, a key player in blood coagulation, represents an acute clinical response associated with an increased risk of venous thromboembolism [14]. With a blood half-life of approximately 12 h, FVIII serves as an indicator for detecting the presence of thrombi in the bloodstream or the likelihood of PE. To date, the detection of FVIII has primarily relied on classical immunoaffinity-based methods such as Western blot and ELISA. In addition, several techniques have been developed for detecting FVIII, including attenuated total reflection Fourier transform infrared spectroscopy [15] and electrochemical biosensors [16]. On the other hand, troponin I is a striated muscle protein known in the heart as cardiac TnI (cTnI). Levels of cTnI remain unaffected by skeletal muscle injuries such as rhabdomyolysis or multiple traumas. However, in cases of myocardial damage, such as acute MI or acute coronary disease, cTnI levels rise accordingly. This elevation typically begins 3 to 6 h after the onset of acute MI, peaking at 12 to 16 h post-attack. Given its tissue specificity, cTnI serves as a reliable marker for myocardial damage. Currently, various techniques are available for detecting cTnI, including enzyme-linked immunosorbent assay (ELISA) [17], surface plasmon resonance (SPR) [18,19], colorimetry [20], electrochemiluminescence immunoassay [21,22], electrochemical sensing [23,24,25], and photoelectrochemical immunoassay [26,27].

In this study, the mixture of anti-FVIII antibody and anti-cTnI antibody were immobilized on the PEMI biosensor to facilitate the detection of FVIII and cTnI via antigen–antibody interactions. Silk fibroin (SF) and graphene oxide (GO) were employed as immobilization matrices for both antibodies. SF is a natural biopolymer derived from silkworm silk, known for its exceptional biocompatibility. It is commonly used for the immobilization of enzymes or antibodies through methods such as covalent immobilization, adsorption, encapsulation, or cross-linking [28]. In addition, GO, an oxidized form of graphene featuring oxygen-containing groups like epoxide, carboxyl, and hydroxyl functional groups on its carbon surface [29], was generally utilized as a substrate coating in sensor applications [30,31]. Since SF can react with carboxylic acid (-COOH), GO was used as an indispensable material for surface modification in this study. This biosensor is a disposable, label-free chip that requires only microliter-level sample volumes. It is capable of detecting both PE and MI antigens, rather than being limited to a single antigen type. It differentiates between PE and MI by utilizing specific upper and lower thresholds. Furthermore, it can evaluate the severity of these conditions. The timely differentiation of PE or MI is essential for administering appropriate treatment.

## 2. Materials and Methods

### 2.1. Materials

The materials used for the surface modification of the PEMI biosensor included GO and SF. GO was synthesized from graphite powder using a modified Hammer’s method, with materials such as graphite (<20 micron synthetic, purchased from SIGMA-Aldrich (St. Louis, MO, USA)), H_2_SO_4_, Na_2_SO_4_, KMnO_4_, H_2_O_2_, and a dialysis membrane. Additionally, SF (5% solution) was obtained from Advanced BioMatrix (San Diego, CA, USA).

The essential biological materials included FVIII (powder, 10 IU/mL, purchased from ProSpec (Rehovot, Israel)), recombinant human cTnI protein (0.25 mg/mL solution, purchased from Abcam (Cambridge, UK)), recombinant anti-FVIII antibody (1.32 mg/mL solution, purchased from SinoBiological (Beijing, China)), and anti-cTnI antibody (2 mg/mL solution, purchased from Abcam (Cambridge, UK)).

In addition, phosphate-buffered saline (PBS) with a pH of 7.4, prepared by dissolving Na_2_HPO_4_, KH_2_PO_4_, NaCl, and KCl in deionized (DI) water, was used as a necessary reagent for bioassays. The solution was then mixed to create dilute buffer and washing buffer.

### 2.2. Chip Design, Fabrication, and Biofunctionalization

**Figure 1:**
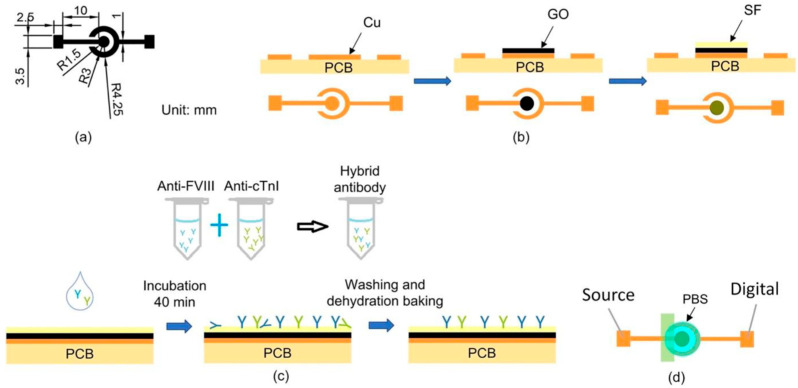
(**a**) The design and dimensions of the biosensor. (**b**) The surface modification process. (**c**) The biofunctionalization process. Different colors represent different antibodies. (**d**) The electrical measurement process.

The PEMI biosensor comprised a working electrode (WE) and a counter/reference electrode (CE/RE), as shown in Figure 1a. This biosensor was utilized for mapping current-to-potential of analytes. A printed circuit board (PCB) served as the substrate for fabricating the PEMI biosensor, with its copper film etched to form the electrode structure using conventional PCB processing technology. Subsequently, the copper working electrode underwent modification with a layer of GO to introduce carboxylic acid (-COOH) groups for SF bonding. The surface modification process is depicted in Figure 1b and detailed as follows: Initially, 0.11 g of GO powder was dispersed in 9.89 mL of deionized water (DI) via ultrasonic vibration until achieving a colloid of 1 wt% GO solution. Then, 8 μL of 1 wt% GO aqueous solution was drop-cast onto the working electrode and dried in a 37.4 °C oven for 1 h to form a GO thin film. Subsequently, 5 μL of diluted SF aqueous solution was deposited onto the GO-coated electrode and dried in the oven at 37.4 °C for 2 h until the SF membrane turned transparent with a yellow hue. Following this, 300 μL of PBS was used to wash the electrode surface to remove loosely bonded SF. The surface was further rinsed with 300 μL of DI water to eliminate residual salt from the PBS, resulting in the SF/GO-modified electrode.

After surface modification, the biofunctionalization process was conducted to immobilize antibodies onto the biosensor, as shown in Figure 1c. The anti-FVIII and anti-cTnI antibodies were individually diluted with PBS at a ratio of 1 μL of antibody to 1000 μL of PBS. This results in final concentrations of 1.32 μg/mL for anti-FVIII and 2 μg/mL for anti-cTnI. The mixtures were thoroughly mixed using an oscillator for 15 min to achieve homogeneous dilution. For subsequent bioassays, the two antibody diluents were combined in a 1:1 ratio to form a mixture of antibody solution. The mixture was gently shaken in an oscillator for 15 min. Next, 8 μL of the antibody mixture was applied to the surface of the SF/GO-modified electrode, followed by incubation in a 37 °C oven for 40 min. The biosensor was then rinsed successively with 300 μL of PBS Tween-20 buffer (PBST) and 300 μL of DI water to ensure complete removal of incompletely bound antibodies, thereby preventing issues in subsequent experiments. Finally, dehydration baking was performed in a 37 °C oven for 10 min to eliminate moisture from the electrode surface. The biofunctionalization process involved immobilizing a mixture of antibodies, including anti-FVIII and anti-cTnI, on the SF/GO-modified electrode surface for binding to FVIII and cTnI antigens, enabling the detection of PE and MI diseases.

### 2.3. Measurement Procedure

To detect and identify PE or MI in a patient, a drop of analyte (typically the patient’s plasma) was applied onto the biofunctionalized PEMI biosensor. The biosensor was then incubated in a 37 °C oven for 30 min and thoroughly rinsed. Subsequently, an insulating tape was utilized to cover the portion of the biosensor lacking surface modification, as depicted in Figure 1d, and electrical measurements were conducted using a high-accuracy electric meter Keithley 2614B (Keithley Instruments, Solon, OH, USA). Prior to measurement, 50 μL of 10 mM PBS (pH = 7.4) was added to the electrode. Linear sweep voltammetry (LSV) was performed with a voltage range of −1 to 0 V in 0.01 V intervals.

## 3. Results and Discussion

### 3.1. Results of Surface Modification and Biofunctionalization Process

**Figure 2:**
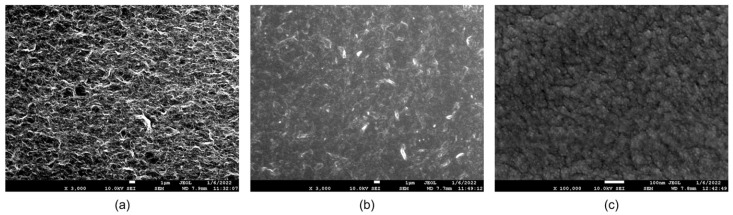
The SEM images after surface modification and biofunctionalization process: (**a**) The modified GO surface in the form of a uniformly distributed mesh. (**b**) SF coating in a semi-translucent and flat form. (**c**) Antibodies adsorbed as fine spheroids.

The surface morphologies of the PEMI biosensor were examined using a JOEL JSM-7600F field emission scanning electron microscope (FE-SEM) to observe the changes occurring during the steps of surface modification and biofunctionalization. The surface modification involved the application of a 1 wt% GO aqueous solution onto the copper electrode. As shown in Figure 2a, the modified GO surface exhibited a uniformly distributed mesh structure, distinct from the original flakes of GO powder. This homogeneity was achieved after one week of ultrasonic mixing, enabling even distribution on the working electrode surface during coating and successful grafting with subsequent layers. After SF modification, the surface appeared semi-translucent and flat, as shown in Figure 2b, indicating SF penetration into the pores of the GO surface and tight adhesion to the GO substrate. Upon biofunctionalization, the surface exhibited fine spheroidal structures after antibody adsorption, as shown in Figure 2c.

The observed variations in the working electrode surface, as shown in the SEM images, suggest the successful completion of both surface modification and biofunctionalization processes.

Additionally, in the biofunctionalization steps of this study, glutaraldehyde, 1-ethyl-3-(3-(dimethylamino)propyl) carbodiimide hydrochloride (EDC), N-hydroxysuccinimide (NHS), and similar agents were not used to form cross-links or covalent bonds. Instead, the antibodies were immobilized through adsorption.

### 3.2. Detection Performance by Single Antibody

The detection performance of the PEMI biosensor biofunctionalized with only a single antibody was first investigated. Based on the biofunctionalized antibody, these biosensors were labeled anti-FVIII/SF/GO and anti-cTnI/SF/GO, respectively.

**Figure 3:**
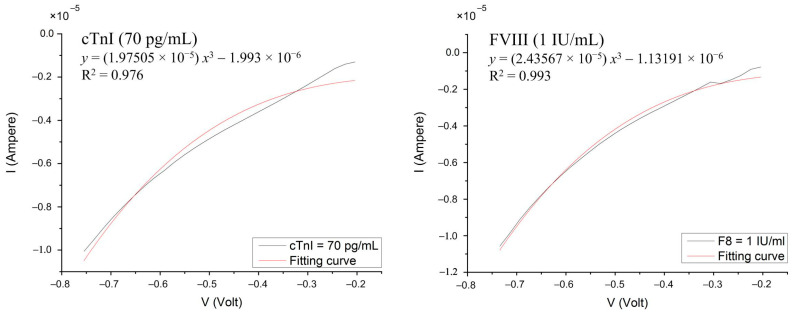
The measured current–voltage (I–V) curves using a high-accuracy electric meter (Keithley 2614B). The curvature of the fitted (red) curve is used as a judgment index.

To evaluate the anti-FVIII/SF/GO biosensor, the concentration range of antigen FVIII was set from 0.5 to 2.5 IU/mL, with a concentration interval of 0.25 IU/mL. Similarly, for testing the anti-cTnI/SF/GO biosensor, the concentration range of cTnI was set from 30 to 210 pg/mL with a sampling interval of 20 pg/mL. Each diluted antigen solution (8 μL) was coated onto the corresponding antibody-biofunctionalized biosensor and incubated in a 37 °C oven for 30 min, followed by the aforementioned rinsing procedure. For statistical analysis of the experimental data, the experiments at each concentration were performed in triplicate with electrical measurement. Figure 3 presents the measured current–voltage (I-V) curves for FVIII and cTnI, respectively, over a voltage range from −0.75 V to −0.2 V. Within this voltage range, the measured signals reached stability, and these curves were then fitted by the equation $y=Ax^{3}+B$, where A represents the degree of curvature of the curve. It is a dimensionless quantity. The larger the value of curvature A, the more curved the overall curve is; whereas, the smaller the value of curvature A, the closer the curve is to a straight line. In a physical sense, under an applied sweeping potential, the current measured on a biosensor with a larger curvature A is greater than that measured on a biosensor with a smaller curvature. Additionally, B represents the intersection of the fitted curve and the y-axis when the voltage is 0 V. The value of B might vary from batch to batch of prepared materials, such as GO, and can be used to calibrate the curve.

**Figure 4:**
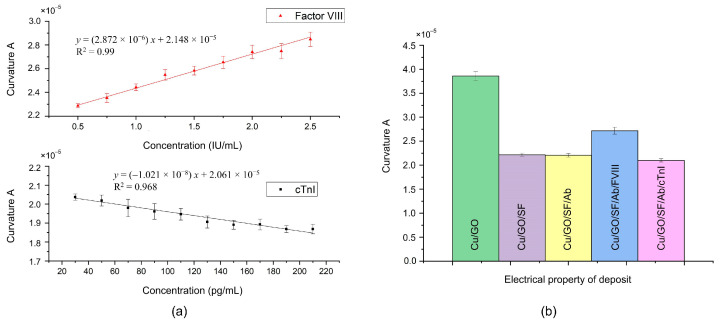
Detection performance by single antibody: (**a**) The curvature A was linearly related to the single antibody concentration of anti-FVIII and anti-cTnI, respectively. (**b**) Electrical measurements of the layer-by-layer deposits on PEMI biosensor.

As shown in Figure 4a, the curvature A exhibits a linear relationship with the concentration of antigen FVIII, while it is inversely linearly proportional to the concentration of antigen cTnI. These observations were further confirmed through electrical measurements of the layer-by-layer deposits, as shown in Figure 4b. Upon deposition of SF onto the surface of GO/Cu, a decrease in curvature A was observed. Due to SF being an insulating film [32], the interfacial electron transfer resistance of the SF/GO/Cu increases, resulting in a decrease in its curvature A. This result also indicates the successful adsorption of SF onto GO/Cu. Subsequent deposition of the antibody on the SF layer, as per the biofunctionalization process, resulted in minimal change in curvature A. However, upon addition of the antigen FVIII to the antibody layer through the incubation process, an increase in curvature A was noted. This result is consistent with that of a previous report [16]. Conversely, the curvature A decreased following the addition of antigen cTnI to the antibody layer. Similar to SF, the interfacial electron transfer resistance of the cTnI/Ab/SF/GO/Cu increases. As the concentration of cTnI increases, its impedimetric response becomes larger [30,33,34], resulting in a smaller curvature and forming a negative slope in Figure 4a.

Different surface modifications are typically necessary to immobilize different antibodies for subsequent bioassays. However, it is noteworthy that SF can immobilize both antibodies (anti-FVIII and anti-cTnI), thereby simplifying the biosensor fabrication process.

### 3.3. Detection Performance by Mixed Antibodies

Subsequently, the mixture of antibodies was investigated. The PEMI biosensor was prepared by mixing equal amounts of the two antibodies using the aforementioned procedure.

**Figure 5:**
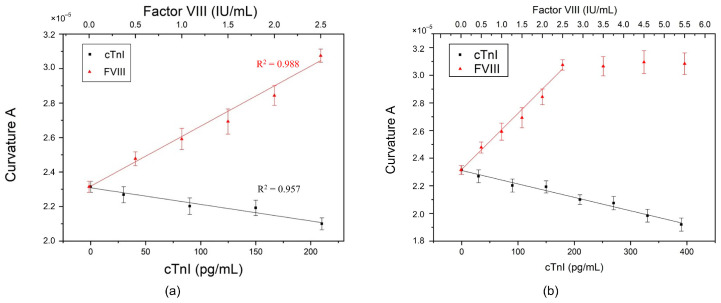
PEMI biosensor with the mixture of antibodies: (**a**) Detection performance. (**b**) Maximum measurable concentration.

In an experiment, a drop of 8 μL of antigen sample (FVIII or cTnI) was titrated onto the biosensor biofunctionalized with the mixture of antibodies. After the incubation process and measurement, the experimental results showed that the curvature A maintained a linear relationship with the concentration of each antigen, i.e., linearly proportional to FVIII but inversely proportional to cTnI, as shown in Figure 5a, and as described by the following regression equations:

(1) $$y=2.932\times 10^{-6}x+2.312\times 10^{-5}\;(\mathrm{for}\;\mathrm{FVIII})$$

and

(2) $$y=-9.604\times 10^{-9}x+2.308\times 10^{-5}\;(\mathrm{for}\;\mathrm{cTnI})$$

where y is the curvature A and x is the concentration. In addition, each linear slope was similar to that obtained with a single antibody.

The results indicated that, when the antibodies were mixed and biofunctionalized, they did not interfere with each other and could still generate electrical changes according to the properties of different antigens in the antibody–antigen interaction. Therefore, in the case of detecting a single antigen, the type and concentration of antigen can be determined by the position of the curvature A. When the specimen sample contains FVIII, the location of the curvature A would be close to the upper line in Figure 5a, indicating a possible risk of PE. Conversely, the position of the curvature A would tend towards the lower line in Figure 5a when the sample contains cTnI, implying a possible risk of MI.

Figure 5b shows the maximum measurable concentration of the PEMI biosensor. For FVIII, the maximum measurable concentration is 2.5 IU/mL; beyond this concentration, the curvature A saturates and no longer changes. The red linear portion in Figure 5b corresponds to the red line for FVIII in Figure 5a. However, for cTnI, the concentration remains linear up to approximately 400 pg/mL.

### 3.4. Detection Performance of Mixed Antibodies vs. Coexisting Antigens

The coexistence of FVIII and cTnI in serum prompted an in-depth investigation in this study on whether the PEMI biosensor can effectively detect these coexisting antigens. Therefore, a mixture of antigens FVIII and cTnI was utilized for drop-casting onto the mixed-antibody PEMI biosensor. Five different concentration combinations of the antigens were tested, as listed in Table 1. A mixture of antigens FVIII and cTnI was utilized for drop-casting onto the mixed-antibody PEMI biosensor.

**Figure 6:**
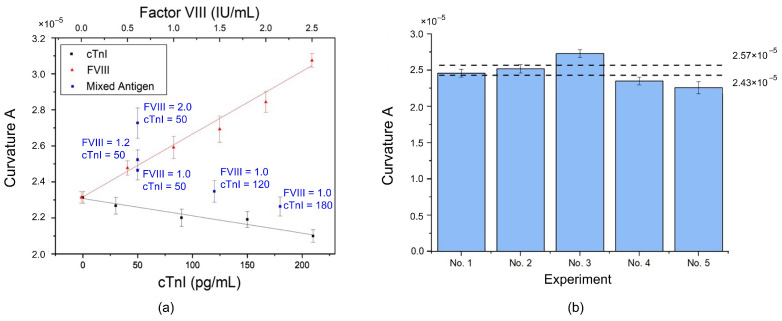
(**a**) The curvatures of coexisting FVIII and cTnI: Inability to distinguish between normal and abnormal conditions. (**b**) Histogram with upper and lower limits to detect PE or MI.

Given that FVIII and cTnI have different units, the curvatures of the experimental data were plotted in Figure 6a based on the unit of cTnI. During electrical measurement, both antigens contributed to the current. Intuitively, titration of the antigen mixture might result in the superposition of measured currents generated by FVIII and cTnI, respectively, leading to a larger curvature A. However, this phenomenon did not occur.

Furthermore, the curvatures of the experimental data scatter between or outside the two linear regression lines of FVIII and cTnI. There was no obvious rule to determine whether PE or MI could be identified using the curvature data points. Thus, a new judgment method was required. In the human body, the normal concentrations of FVIII and cTnI are approximately 1.0±0.5 IU/mL [35] and 30–80 pg/mL [24], respectively. Therefore, the concentration setting in experiment no. 1 (FVIII = 1.0 IU/mL and cTnI = 50 pg/mL) falls within the normal range. The curvature A obtained from experimental measurement was $2.46\times 10^{-5}$, while the calculated curvature value using the regression equations was $4.86518\times 10^{-5}$. Thus, there was a difference of $2.40\times 10^{-5}$ between the actual experimental datum and the calculated regression value. For the remaining experiments, this difference was subtracted from the calculated regression value to estimate curvature A, i.e., γ=β−δ, where β is the calculated curvature A using the regression equations, δ is the difference of $2.40\times 10^{-5}$, and γ denotes the estimated curvature A. Moreover, the estimation error can be calculated by:

(3) $$\varepsilon =\frac{\left|\alpha -\gamma \right|}{\alpha }\times 100,$$

where α is the curvature A obtained using the experimental measurement. As shown in Table 1, the estimated value γ of curvature A had a small error with its corresponding experimental datum α. For experiments no. 2 to no. 5, the errors were all less than 3.5%. Clearly, the difference value δ can be used to estimate curvature A from the calculated regression value without conducting experiments. Additionally, it can be used to establish the limits for judging PE or MI.

Based on the difference value δ obtained from experiment no. 1, the calculated normal maximum and minimum curvature values were $2.43\times 10^{-5}$ and $2.57\times 10^{-5}$, respectively. These two values were adopted as the upper and lower limits for detecting PE and MI. Subsequently, the five sets of experimental curvature data were plotted in a histogram, as shown in Figure 6b. The two dotted lines represent the upper and lower limits, respectively. Any curvature A above the upper limit can be identified as PE, while any curvature A below the lower limit indicates MI. The farther the curvature A is from the upper/lower limit, the more severe the condition.

The curvature A of experiment no. 1 falls between the upper and lower limits, indicating that the concentrations of FVIII and cTnI are normal. This result is expected because the upper/lower limits were derived from the difference δ calculated from experiment no. 1. Similarly, the curvature A of experiment no. 2 also lies within the limits, as the concentrations (FVIII = 1.2 IU/mL and cTnI = 50 pg/mL) are within the normal range. However, the curvature A of experiment no. 3 exceeds the upper limit due to the high concentration of FVIII (2.0 IU/mL). Similar results were observed for cTnI, but the curvatures were below the lower limit.

## 4. Conclusions

Pulmonary embolism and myocardial infarction affect millions of people worldwide each year, posing a significant risk of mortality. Their clinical manifestations, such as chest pain, dyspnea, night sweats, and fainting, often overlap, making prompt diagnosis challenging for healthcare professionals. In this study, the proposed PEMI biosensor offers a solution by enabling the identification and quantitative detection of PE or MI, providing valuable time for doctors to administer appropriate treatments. This biosensor operates on a label-free basis, utilizing linear sweep voltammetry measurement. Silk fibroin and graphene oxide were employed in the immobilization process of both anti-FVIII and anti-cTnI on the working electrode of the PEMI biosensor. The fabrication process is simple, and the cost is minimal, rendering it disposable. Additionally, the established detection upper and lower limits facilitate the accurate identification and quantitative detection of PE and MI. Thus, the PEMI biosensor holds great promise for future point-of-care diagnostics.

## Figures and Tables

**Table 1:** Experiments with five different concentration combinations of FVIII and cTnI.

Exp.	Exp. Setting ^1^		Curvature *A*			Error (ε)
No	FVIII	cTnI	Exp. Data (α) ^2^	Reg. Calc. (β) ^3^	Estimate (γ)	
1	1.0	50	2.46 × 10^−5^	4.86518 × 10^−5^		
2	1.2	50	2.52 × 10^−5^	5.01178 × 10^−5^	2.61 × 10^−5^	3.4%
3	2.0	50	2.73 × 10^−5^	5.15838 × 10^−5^	2.76 × 10^−5^	1.1%
4	1.0	120	2.35 × 10^−5^	4.79795 × 10^−5^	2.40 × 10^−5^	2.1%
5	1.0	180	2.26 × 10^−5^	4.74033 × 10^−5^	2.34 × 10^−5^	3.3%

^1^ Concentration settings for experiments. ^2^ The curvature A obtained from experimental measurement. ^3^ The calculated curvature A using the regression equations.

## Data Availability

The data presented in this study are available upon request from the corresponding author.
